# High-resolution protein modeling through Cryo-EM and AI: current trends and future perspectives – a review

**DOI:** 10.3389/fmolb.2025.1688455

**Published:** 2025-10-23

**Authors:** Gnanaprakash Jeyaraj, Anith Kumar Rajendran, Kuppusamy Sathishkumar, Bader O. Almutairi, Aanand Vadivelu, Santosh Chokkakula, Yanyang Tu, Weimin Xie

**Affiliations:** 1 Saveetha Medical College and Hospital, Saveetha Institute of Medical and Technical Sciences, Chennai, Tamil Nadu, India; 2 Department of Genetic Engineering, SRM Institute of Science and Technology, Kattankulathur, Tamil Nadu, India; 3 Department of Biotechnology, Rathinam College of Arts and Science, Coimbatore, Tamil Nadu, India; 4 Department of Zoology, College of Science, King Saud University, Riyadh, Saudi Arabia; 5 Department of Microbiology, Chungbuk National University College of Medicine and Medical Research Institute, Cheongju, Chungbuk, Republic of Korea; 6 Science Research Center, Huizhou Central People’s Hospital, Huizhou, Guangdong, China; 7 Department of Gynecology, Affiliated Hengyang Hospital of Hunan Normal University & Hengyang Central Hospital, Hengyang, Hunan, China

**Keywords:** cryo-electron microscopy, artificial intelligence, protein structure prediction, AlphaFold, structural biology

## Abstract

The structural elucidation of proteins is fundamental to understanding their biological functions and advancing drug discovery. Recent breakthroughs in cryo-electron microscopy (cryo-EM) and artificial intelligence (AI)-based structure prediction have revolutionized protein modeling by enabling near-atomic resolution visualization and highly accurate computational predictions from amino acid sequences. This review summarizes the latest advances that have transformed protein structural biology from a predominantly structure-solving endeavour to a discovery-driven science. We discuss the complementary roles of cryo-EM and AI, including developments in direct electron detectors, advanced image processing, and deep learning algorithms exemplified by AlphaFold 2 and the emerging AlphaFold 3. These technologies facilitate detailed insights into challenging protein targets such as membrane proteins, flexible and intrinsically disordered proteins, and large macromolecular complexes. Furthermore, we highlight applications of integrative approaches in drug design, enzymatic mechanism elucidation, and functional predictions, illustrated by examples including hemoglobin, which demonstrates both the strengths and current limitations of AI–cryo-EM integration, and cytochrome P450 enzymes, where AlphaFold predictions have been combined with cryo-EM maps to explore conformational diversity. The review also addresses ongoing challenges and promising future directions for integrating experimental and computational methods to accelerate the exploration of protein structure–function relationships, ultimately impacting biomedical research and therapeutic development.

## Graphical Abstract

**Figure:**
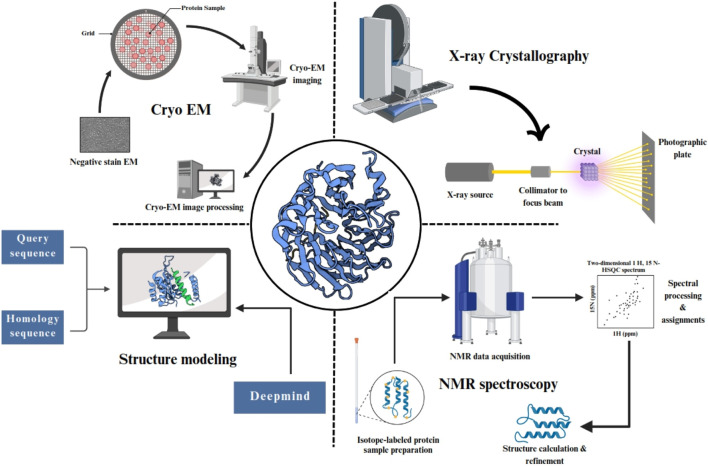

## Highlights

- Cryo-EM and AI enable accurate, high-resolution modeling of diverse protein structures.
- AI tools like AlphaFold drive rapid protein structure prediction and functional discovery.
- Integrative approaches resolve structures of membrane proteins and flexible assemblies.
- Structure insights guide drug design and disease research for targets like viral and amyloid proteins.
- Advances pave the way for discovery-driven protein science and therapeutic innovation.

## 1 Introduction

Biological macromolecules proteins, nucleic acids, and polysaccharides are essential components of life, driving fundamental processes such as DNA replication, enzyme activity, and cellular communication. For example, hemoglobin transports oxygen in the blood, while DNA polymerases ensure the accurate copying of genetic material during cell division (Kornberg Arthur, 1992; PERUTZ et al., 1960). Polysaccharides like cellulose provide structural support to plant cell walls, and glycosaminoglycans in the extracellular matrix help regulate tissue flexibility and cell signaling (Cosgrove, 2005; Hynes and Naba, 2012). The function of these macromolecules is directly linked to their three-dimensional (3D) structures, which determine their folding, interactions, and biological roles (Dill and MacCallum, 2012). Understanding these structures extends beyond academic interest, playing a pivotal role in addressing global health, energy, and sustainability challenges.

Structural biology is dedicated to elucidating the architectural design of biological macromolecules, aiding in the comprehension of their functions and the development of new drugs, biomaterials, and industrial enzymes. The field has advanced dramatically since the first structure of myoglobin was determined using X-ray crystallography (Kendrew et al., 1958). Technological innovations from early crystallography to contemporary cryo-electron microscopy (cryo-EM) and artificial intelligence (AI)-driven structure prediction have significantly enhanced our ability to visualize and understand molecular structures (Jumper et al., 2021; Nogales, 2016).

X-ray crystallography has long been a cornerstone of structural biology, helping scientists determine high-resolution structures of countless proteins, nucleic acids, and their complexes. For example, this technique was used to solve the structure of the ribosome, a large molecular machine responsible for protein synthesis, revealing intricate details about how it functions (Yusupov et al., 2001). However, X-ray crystallography has its limitations, particularly when studying large, flexible, or membrane-bound macromolecules that are difficult to crystallize (Caffrey, 2015). Despite these challenges, it remains a vital tool in structural biology, as seen in its role in identifying the structure of the SARS-CoV-2 main protease, a key target for antiviral drug development (Zhang et al., 2020).

Nuclear magnetic resonance (NMR) spectroscopy emerged as a valuable complementary technique, allowing researchers to study macromolecules in solution and observe their dynamic behavior. NMR has been particularly useful in analyzing small to medium-sized proteins, such as the oncogenic protein KRAS, which plays a crucial role in cancer signaling pathways (Ostrem et al., 2013). However, this technique has its own limitations it struggles to analyze larger macromolecular complexes or membrane proteins due to their complexity and size (Wüthrich, 2001).

The introduction of cryo-electron microscopy (cryo-EM) has transformed structural biology by overcoming many of the limitations of traditional techniques. Cryo-EM allows scientists to visualize large macromolecular complexes and membrane proteins at near-atomic resolution without requiring crystallization. The ability to determine atomic structures from patient-derived or native biological samples first demonstrated in studies such as Fitzpatrick et al. illustrates structural biology’s transformation into a powerful discovery tool capable of generating novel hypotheses and enabling translational advances directly from human disease material (Fitzpatrick et al., 2017). A major breakthrough was the determination of the structure of the TRPV1 ion channel, which revealed how this protein detects heat and pain (Liao et al., 2013). Further advancements, such as direct electron detectors and sophisticated image processing algorithms, have significantly improved the resolution and applicability of cryo-EM, making it a crucial tool in modern structural biology (Kühlbrandt, 2014). A pivotal breakthrough underlying the cryo-EM “resolution revolution” was the introduction of direct electron detection cameras (X. Li et al., 2013). These detectors provide dramatically improved signal-to-noise ratios, accurate electron event counting, and rapid frame rates, enabling correction of beam-induced motion and unlocking near-atomic resolution for previously intractable targets. The landmark structure of the TRPV1 ion channel, for example, was only attainable with these new generation detectors (Liao et al., 2013), which have since become standard across high-resolution cryo-EM studies.

At the same time, computational methods have become essential in structural biology. The rise of AI-driven algorithms, such as AlphaFold and RoseTTAFold, has revolutionized protein structure prediction, allowing scientists to determine structures with remarkable accuracy based solely on amino acid sequences. For example, AlphaFold successfully predicted the structure of the orphan protein ORF8 from SARS-CoV-2, providing crucial insights into its potential role in immune evasion (Gordon et al., 2020; Varadi et al., 2022). These advancements have not only accelerated the pace of structural discovery but have also made structural information more accessible to researchers worldwide, enabling a deeper understanding of molecular biology (Baek et al., 2021).

The combination of experimental and computational approaches has significantly broadened the field of structural biology, allowing researchers to study macromolecules in their natural environments and observe their dynamic structural changes. Techniques such as time-resolved crystallography, small-angle X-ray scattering (SAXS), and integrative modeling have provided groundbreaking insights into the flexible and dynamic nature of biological macromolecules. For instance, time-resolved studies of the photosynthetic reaction center have uncovered the intricate sequence of electron transfer events during photosynthesis (Kern et al., 2013). Similarly, integrative modeling has been used to reconstruct the structure of the nuclear pore complex, a massive molecular assembly responsible for regulating the transport of molecules between the nucleus and the cytoplasm (Kim et al., 2018). In this review, we synthesize recent advances in cryo-EM and AI with a focus on improvements in resolution, heterogeneity analysis, and automated model building. We also discuss how AI-based predictors such as AlphaFold2 are being integrated into cryo-EM workflows to expand their impact. By emphasizing the intersection of these fields, we highlight methodological innovations that are shaping structural biology today.

## 2 Foundational methods in structural biology

Structural biology has historically relied on three primary techniques: X-ray crystallography, nuclear magnetic resonance (NMR) spectroscopy, and electron microscopy (EM). These methods continue to be indispensable, despite recent advancements in cryo-EM and AI-driven structure prediction.

### 2.1 X-ray crystallography

**FIGURE 1:**
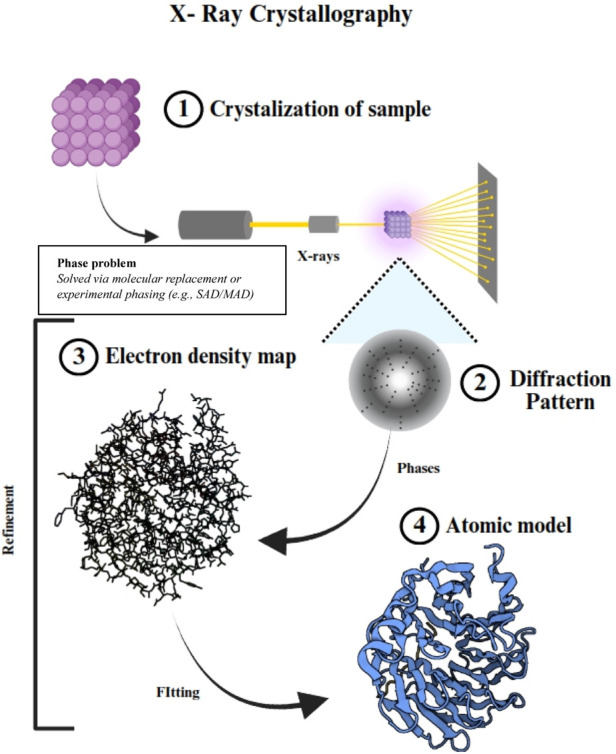
Schematic representation of the X-ray crystallography process for protein structure determination. (1) The sample undergoes crystallization to form an ordered lattice. (2) The crystal is exposed to X-ray beams, generating a diffraction pattern that provides crucial structural information. Note: The “phase problem” arises here, as phase information is not captured in the diffraction data and must be recovered through methods such as molecular replacement or experimental phasing (e.g., SAD/MAD). (3) Computational methods are used to derive an electron density map from the diffraction data, incorporating phase determination techniques. (4) The final atomic model is built and refined by fitting atomic coordinates into the electron density map, resulting in a high-resolution structural representation of the macromolecule.

Since its emergence in the 1950s, X-ray crystallography has been a cornerstone of structural biology, enabling the determination of high-resolution structures of proteins, nucleic acids, and their complexes. By analyzing the diffraction patterns of X-rays passing through crystallized macromolecules, scientists can generate electron density maps to construct atomic models (Drenth J, 2007). Innovations such as microfocus X-ray beams and serial crystallography has expanded its applicability, facilitating the study of smaller crystals and transient molecular states. For example, time-resolved serial femtosecond crystallography (TR-SFX) at X-ray free-electron lasers (XFELs) has provided insights into the catalytic cycle of cytochrome c oxidase, shedding light on electron and proton transfer mechanisms (Kern et al., 2013). Recent innovations in high-throughput screening and automated data processing further illustrate the method’s ongoing relevance and adaptability (Smith et al., 2023). Schematic representation of the X-ray crystallography process for protein structure determination shown in Figure 1.

Another major advancement in membrane protein crystallography was the determination of the β2-adrenergic receptor structure while bound to its agonist using lipidic cubic phase (LCP) crystallization. This study not only provided a high-resolution view of G protein-coupled receptor (GPCR) signaling but also paved the way for the structural characterization of other membrane proteins, including the adenosine A2A receptor and the serotonin receptor (Rasmussen et al., 2011).

X-ray crystallography has also played a crucial role in drug discovery, particularly in antiviral therapy development. For example, the structure of the SARS-CoV-2 main protease (Mpro), determined through crystallography, revealed the enzyme’s active site and enabled the design of inhibitors such as nirmatrelvir, a drug effective in treating COVID-19 (Zhang et al., 2020). Additionally, crystallography has been instrumental in understanding enzyme mechanisms, such as the DNA-cleaving activity of CRISPR-Cas9. The structure of CRISPR-Cas9 in complex with guide RNA and target DNA provided a detailed molecular blueprint for genome editing, revolutionizing biotechnology and medicine (F. Jiang et al., 2015).

Despite its contributions, X-ray crystallography faces challenges in crystallizing large, flexible, or membrane-bound macromolecules. However, continuous advancements in crystallization techniques and computational modeling ensure its enduring significance. Detailed technical advances and their implications are discussed in Section 3.

### 2.2 Nuclear magnetic resonance (NMR) spectroscopy

NMR spectroscopy enables the study of macromolecules in solution, providing insights into their structural dynamics, interactions, and conformational changes. Unlike X-ray crystallography, NMR does not require crystallization, making it particularly useful for analyzing small to medium-sized proteins (Cavanagh et al., 2007). Traditionally, NMR’s strengths have been in resolving the structures and dynamic properties of small to medium-sized proteins (generally <40 kDa), although advances in isotope labeling and high-field instrumentation have gradually extended these boundaries (Wüthrich, 2001).

Solid-state NMR has also emerged as a powerful tool for studying membrane proteins and amyloid fibrils. For example, the structure of the amyloid-β fibril, a key hallmark of Alzheimer’s disease was determined using solid-state NMR, revealing its cross-β architecture and shedding light on the molecular basis of amyloid formation (Tycko, 2011).

NMR is widely used to study protein-ligand interactions, particularly in drug discovery. One notable example is the characterization of the interaction between the oncogenic protein KRAS and its inhibitors. NMR studies have provided important insights into how KRAS undergoes conformational changes upon inhibitor binding, aiding the development of more effective cancer therapies (Ostrem et al., 2013). Advances in high-field NMR have improved resolution and sensitivity, extending its applicability to larger proteins. NMR spectroscopy has played a pivotal role in characterizing intrinsically disordered proteins (IDPs), which lack stable tertiary structures yet perform critical regulatory functions. Notable examples include α-synuclein, implicated in Parkinson’s disease (Burré et al., 2014), and the tumor suppressor p53, whose disordered regions modulate DNA binding and partner interactions (Krois et al., 2018). Recent developments in NMR methodology have further expanded its utility to mammalian mega-complexes and large IDP-containing systems (Joerger and Fersht, 2008).

Despite these advances, NMR remains limited when analyzing large macromolecules due to signal overlap, reduced resolution, and the need for isotopic labeling. Ongoing developments in NMR instrumentation and computational analysis continue to enhance its potential in structural biology.

### 2.3 Electron microscopy (EM)

The landscape of electron microscopy has dramatically evolved in the past decade. While conventional EM (using negative staining and photographic detection) was foundational, it offered limited resolution and structural detail. The so-called “resolution revolution” triggered by the introduction of direct electron detectors and advanced processing algorithms (X. Li et al., 2013) has transformed cryo-EM into a high-resolution, widely adopted tool. Thus, our review focuses on cryo-EM as it is used in current structural biology, and references to “conventional EM” are restricted to historical perspective or legacy techniques. Traditional EM has provided low-resolution visualizations of macromolecular complexes and cellular structures. Recent technological advancements, particularly in cryo-EM, have extended its capabilities, allowing high-resolution imaging of biological macromolecules in near-native states. Negative staining EM has been useful in studying macromolecular assemblies, including ribosomes and viral capsids (Frank, 2006). The development of cryo-EM has transformed the field, providing near-atomic resolution images without requiring crystallization (Kühlbrandt, 2014). Conventional EM, in fact, set the stage for cryo-EM, and in its present form, has reshaped the face of structural biology forever. Early studies with EM of the ribosome produced low-resolution maps, paving the path for future high-resolution cryo-EM structures and revealing molecular detail in protein synthesis (Yusupov et al., 2001). EM played a key role in studying virus morphology, including Ebola and influenza virus. The glycoprotein structure of Ebola, determined with EM, guided vaccine development through its demonstration of the molecular mechanism of virus entry into host cells (J. E. Lee et al., 2008). EM has also helped visualize structures of cellular organelles, including mitochondrial and endoplasmic reticulum structures, and shed light on their function and organization. For example, EM studies of mitochondrial structures revealed the complex arrangement of cristae, inner membrane folds in which cellular respiration occurs (Frey and Mannella, 2000). Conventional EM, despite its success, suffers from poor resolution, generally in the 10–20 Å range, and difficulty in preparing samples for analysis. With cryo-EM and new EM technology, many of these challenges have been overcome, and a new era in structural biology has begun.

Recent years have witnessed the incorporation of novel instrumentation, automation, and computational approaches that substantially expand the capabilities of these foundational methods. Transformative advances including time-resolved crystallography, enhanced NMR techniques, and integration of artificial intelligence are discussed in detail in Section 3.

## 3 Recent advances in structural characterization

Building on the foundational principles introduced earlier, this section highlights recent breakthroughs in cryo-EM, AI-driven prediction, and hybrid modeling that have expanded the capabilities of structural biology beyond conventional limits.

### 3.1 Cryo-electron microscopy (Cryo-EM)

**FIGURE 2:**
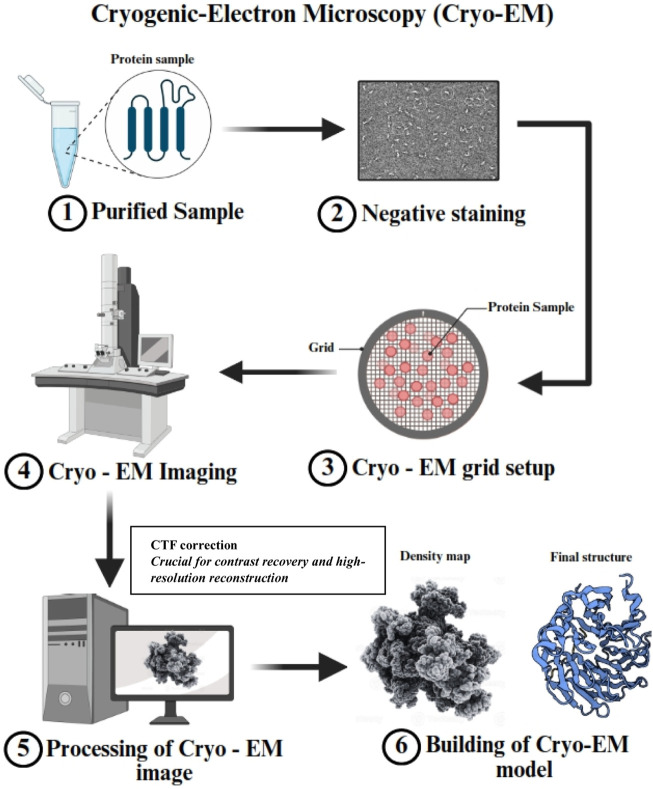
This schematic illustrates the Cryo-EM process for protein structure determination. (1) The purified protein sample is prepared for analysis. (2) Negative staining is performed for initial visualization and sample quality assessment. (3) The protein sample is applied to a Cryo-EM grid for vitrification, ensuring structural preservation. (4) The grid is imaged using a Cryo-EM microscope under cryogenic conditions. (5) Computational processing of Cryo-EM images is performed to generate a high-resolution density map. Note: This step includes essential Contrast Transfer Function (CTF) correction to restore contrast and improve the accuracy of structural reconstruction. (6) The final Cryo-EM structural model is built based on the density map, revealing atomic-level details of the macromolecule.

Cryo-electron microscopy (cryo-EM) is a cutting-edge technology in structural biology, providing visualization of membrane proteins and large macromolecular complexes at near-atomic resolution in their native conformations, without the need for crystallization. Breakthroughs in direct electron detectors, high-performance image processing algorithms, and sample preparation methodologies have driven this revolution (Liao et al., 2013). Unlike traditional techniques, cryo-EM allows the study of proteins in their native conformations, preserving their conformational flexibility and dynamic behavior. This makes cryo-EM particularly beneficial for studying challenging targets such as membrane proteins, large complexes, and intrinsically disordered proteins. Figure 2 schematically illustrates the Cryo-EM process for protein structure determination.

One of the most significant breakthroughs in cryo-EM has been its application to ribosome structures. Ribosomes, complex and dynamic molecular machines responsible for protein synthesis, have long been challenging to study using traditional techniques. Notably, the landmark cryo-EM study by Fitzpatrick et al. provided the first high-resolution structures of tau filaments extracted directly from human Alzheimer’s disease brain tissue, revealing disease-specific filament folds in patient-derived samples for the first time (Fitzpatrick et al., 2017). This breakthrough exemplifies how advances in structural biology have enabled direct molecular discovery in clinically relevant contexts, allowing new hypotheses and therapeutic avenues to emerge from human-derived materials rather than model systems alone. In recent years, cryo-EM has resolved several functionally informative states of the human mitochondrial ribosome, including high-resolution structures with translational factors such as EF-G1 that reveal tRNA translocation conformations (Koripella et al., 2020), more recent pre-initiation mtSSU complexes elucidating initiation factor interactions (Hilander et al., 2024), and a 2.2 Å mitoribosome structure with cofactors and modified tRNAs providing atomic detail of its active components (Singh et al., 2024). These advances deepen understanding of mitochondrial protein synthesis, its dysfunction in disease, and potential structural targets for therapeutic intervention.

Cryo-EM has also facilitated the characterization of membrane proteins, which are notoriously resistant to crystallization. In 2022, a breakthrough study determined the structure of the P2X7 receptor, a key mediator of inflammation and immunity, using cryo-EM (McCarthy et al., 2019). The study revealed the receptor’s activation mechanism, providing a molecular blueprint for the development of anti-inflammatory therapies. Similarly, cryo-EM has been used to explore the capsids of viruses such as SARS-CoV-2 and Zika virus. In 2023, cryo-EM was used to determine the capsid structure of the monkeypox virus, offering new insights into its assembly and potential antiviral therapy targets (Xu et al., 2023).

Resolution remains a critical parameter in cryo-EM, as it determines the level of structural detail that can be extracted from density maps. While crystallographers reserve the term *atomic resolution* for data near 1.2 Å, at which even hydrogen atoms can be visualized (Nakane et al., 2020; Wlodawer and Dauter, 2017), in cryo-EM practice maps around 3 Å are widely regarded as atomic or near-atomic because amino-acid side chains and backbone atoms can be traced with confidence, enabling *de novo* model building (Yip et al., 2020; Zhou, 2011). Statistical analyses of the EMDB show the rapid progress of the field: a decade ago most reconstructions were in the 4–10 Å range, but by 2023–2024 the majority of deposited maps achieved better than 4 Å, with nearly half falling in the 3–5 Å range and record structures approaching 1.2 Å (Iudin et al., 2023; Kleywegt et al., 2024). The interpretability of cryo-EM data is strongly resolution-dependent: at ∼10 Å, only the overall molecular envelope and gross domain organization are visible; at ∼5 Å, α-helices appear as rods and β-sheets as slabs, but side chains are not individually resolved; and at ∼3 Å, side chains are clearly distinguishable and atomic models can be constructed with near-crystallographic accuracy (Rosenthal and Rubinstein, 2015; Zhou, 2011).

Recent advances in AI-driven cryo-EM data processing go well beyond generic neural networks, with the development of specialized tools such as DeepPicker (Wang et al., 2016) and Topaz (Bepler et al., 2019). DeepPicker was among the first to employ deep convolutional neural networks for automatic, accurate particle selection, while Topaz further improved accuracy and speed, enabling robust particle picking even for challenging datasets. Integration of these tools into platforms like cryoSPARC and RELION has streamlined high-throughput structure determination and significantly elevated reconstruction quality.

The establishment of large-scale standardized datasets has been pivotal for advancing AI-based particle picking in cryo-EM. CryoPPP, developed by Dhakal et al. (2023), is the largest expert-curated resource to date, encompassing 9,893 micrographs and more than 400,000 annotated particles across 34 protein datasets. It now serves as a benchmark platform for training and evaluating new particle-picking algorithms under consistent conditions. Building directly on this foundation, CryoTransformer was introduced as a state-of-the-art transformer-based model that employs attention mechanisms to capture long-range dependencies in cryo-EM micrographs (Dhakal et al., 2024). Comprehensive benchmarking on CryoPPP and EMPIAR datasets showed that CryoTransformer achieved F1-scores ranging from 0.65 to 0.85 and consistently produced 3D reconstructions in the 4–6 Å resolution range, outperforming established methods such as Topaz and crYOLO. Together, CryoPPP and CryoTransformer exemplify how standardized datasets and next-generation deep learning architectures are reshaping one of the most labor-intensive steps in cryo-EM image analysis.

The impact of cryo-EM on structural biology cannot be overstated. By enabling the study of previously intractable targets, cryo-EM has democratized structural biology, making it accessible to researchers worldwide. Its ability to determine multiple conformational states has provided unprecedented insights into macromolecular dynamics, revealing molecular processes such as enzyme catalysis, signal transduction, and viral infection. As cryo-EM continues to evolve, it will have an even greater impact on drug development, biotechnology, and our understanding of fundamental biological processes.

While cryo-EM has expanded access to high-resolution structure determination beyond crystallography, significant barriers remain, including high capital and maintenance costs of instrumentation, the need for specialized technical expertise in sample preparation and data collection, and the requirement for substantial computational infrastructure for data processing and reconstruction (Carroni and Saibil, 2016; Cheng et al., 2015). These factors can limit accessibility, particularly for smaller labs or in low-resource settings.

### 3.2 Artificial intelligence (AI) in structure prediction

The development of AI algorithms, such as AlphaFold and RoseTTAFold, has revolutionized structural biology by enabling the prediction of high-accuracy protein structures directly from amino acid sequences. These tools are based on deep learning and leverage large databases of high-quality protein structures to predict 3D structures with remarkable accuracy, often surpassing experimental approaches (Jumper et al., 2021). This has not only accelerated the pace of discovery but has also democratized access to structural information, allowing researchers worldwide to explore the molecular basis of life.

One of the most significant breakthroughs in AI-driven structure prediction occurred in 2021 with the launch of the AlphaFold Protein Structure Database, which contains predicted structures for nearly the entire human proteome. This database serves as a valuable resource for functional annotation and drug discovery (Varadi et al., 2022). For example, AlphaFold’s prediction of the SARS-CoV-2 orphan protein ORF8 provided crucial insights into its role in immune evasion (Gordon et al., 2020). This breakthrough confirmed the potential of AI to drive rapid advancements in structural discovery and therapeutic development.

While AI-based methods such as AlphaFold and RoseTTAFold have revolutionized protein structure prediction, significant limitations remain especially for intrinsically disordered regions (IDRs), large multiprotein complexes, and novel folds. Quantitative benchmarks show that for well-ordered protein domains, AlphaFold2 achieves atomic-level accuracy (RMSD <1.5 Å), but for IDRs and flexible linkers, RMSD often exceeds 5–10 Å and structural predictions can be unreliable or misleading (Robin et al., 2021; Tunyasuvunakool et al., 2021). For example, results from CASP14 and CASP15 showed consistent declines in AlphaFold’s accuracy when modeling highly dynamic regions or novel folds not represented in the training data (Kryshtafovych et al., 2023). Tools for probing intrinsically disordered proteins (IDPs) are advancing rapidly. For example, IDPConformerGenerator generates large and diverse conformational ensembles of disordered states, enabling systematic sampling of backbone and side-chain variation (Teixeira et al., 2022). In parallel, deep-learning-based methods such as ALBATROSS directly predict ensemble-averaged dimensions including radius of gyration, end-to-end distance, and polymer scaling exponents from sequence information (Lotthammer et al., 2024). These computational benchmarks enable comparison of AI-predicted structural models of IDRs. Yet challenges remain: studies have shown that flexible termini (e.g., in p53) are frequently mis-modeled; multi-subunit complexes may omit relevant dynamic conformers; and protein–protein or protein-nucleic acid interfaces in disordered regions are often only partially captured (Evans et al., 2022; Jumper et al., 2021; Ruff and Pappu, 2021). Together, these observations emphasize the need for integrating ensemble-based computational approaches with experimental validation (such as NMR, SAXS, or HDX-MS) to achieve realistic representations of IDPs. Additionally, while AlphaFold’s error metrics (e.g., pLDDT, PAE) provide confidence estimates, they can be misinterpreted, and high scores do not always correlate with biological accuracy, particularly in overfitted or underrepresented regions (Akdel et al., 2022). Addressing these limitations will require improved datasets, better calibration of confidence metrics, and integration with experimental data for challenging targets.

A key factor underlying the success of AlphaFold is its use of multiple sequence alignments (MSAs) to capture evolutionary covariation between residues, which provides constraints for predicting inter-residue distances and orientations. The depth and diversity of the MSA strongly influence predictive accuracy. When large, diverse sets of homologous sequences are available, AlphaFold2 achieves near-atomic accuracy for many protein domains, often with root mean square deviations (RMSDs) of less than 1.5 Å relative to experimental structures (Jumper et al., 2021). However, when only a limited number of homologs can be identified, such as for orphan proteins or rapidly evolving viral proteins, predictive performance drops substantially. Benchmarking in CASP14 and CASP15 consistently showed that proteins with shallow MSAs yielded lower-confidence models, with errors particularly pronounced in flexible loops, novel folds, or intrinsically disordered regions (Akdel et al., 2022; Kryshtafovych et al., 2023). In such cases, AlphaFold’s confidence metrics (pLDDT, PAE) may overestimate structural reliability, and experimental validation or hybrid modeling approaches remain essential. This limitation highlights that AlphaFold’s power is fundamentally coupled to the availability of evolutionary information, and regions of sequence space lacking homologs remain challenging.

**FIGURE 3:**
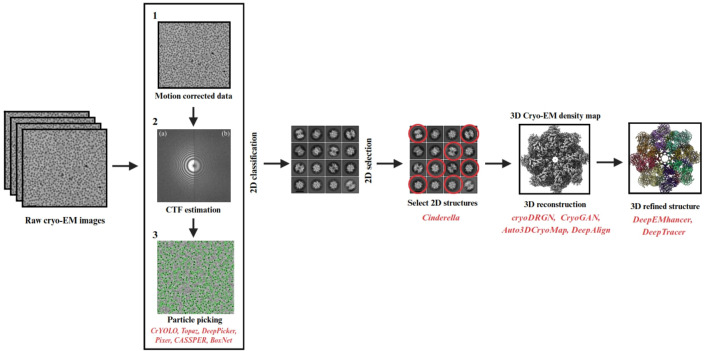
Integration of AI into cryo-EM data processing.

In addition to sequence-based predictors such as AlphaFold and RoseTTAFold, artificial intelligence is increasingly applied directly to cryo-EM data analysis, spanning particle picking, density map interpretation, and heterogeneity reconstruction. At the front end of the workflow, AI-driven particle picking methods such as Topaz, crYOLO, CryoSegNet, and CryoTransformer have greatly improved the accuracy and throughput of identifying particles in noisy micrographs, thereby reducing one of the most labor-intensive bottlenecks in single-particle analysis (Dhakal et al., 2024). For atomic model building, tools like DeepTracer, DeepMainmast, and ModelAngelo leverage convolutional and graph neural networks to trace main-chain coordinates and assign residues directly from cryo-EM density maps, producing models that rival expert-curated reconstructions (Jamali et al., 2024; Terashi et al., 2024). Supporting this, datasets such as Cryo2StructData, comprising over 7,600 labeled experimental cryo-EM density maps, now provide the large-scale training resources needed to train next-generation AI models for reliable automated structure determination (Giri et al., 2024). AI has also advanced heterogeneity analysis, with CryoDRGN, a deep generative network that learns continuous distributions of 3D structures to reveal conformational variability across particle ensembles (Zhong et al., 2021), and CryoFIRE, which applies amortized inference to jointly estimate particle orientations and conformational states, enabling efficient reconstruction of flexible complexes without exhaustive alignment (Levy et al., 2022). Collectively, these developments demonstrate how AI is reshaping cryo-EM pipelines end-to-end, from raw image preprocessing through to final atomic models and ensemble characterization. The role of AI in particle picking, reconstruction, and heterogeneity analysis, since the Figure 3 visually demonstrates exactly that workflow.

Schematic overview showing how deep learning methods are applied across cryo-EM workflows, including motion correction, CTF estimation, particle picking (e.g., crYOLO, Topaz, DeepPicker), 2D/3D classification and selection (e.g., Cinderella), and final map interpretation and model building (e.g., DeepTracer, cryoDRGN). AI tools enhance accuracy, speed, and automation throughout the cryo-EM pipeline.

Another important limitation of current AI-based structure prediction tools is their limited consideration of experimental and environmental conditions. Most methods, including AlphaFold2/3 and RoseTTAFold, generate a single (or dominant) structure based on sequence and evolutionary data, effectively modeling the protein in a “standard” context and neglecting factors such as pH, ionic strength, redox environment, ligand or cofactor presence, and post-translational modifications. However, many proteins adopt different conformations depending on local conditions for example, a protein’s structure at pH 5 can differ substantially from its structure at pH 7 (Jumper et al., 2021; Madhavi Sastry et al., 2013). Addressing these real-world biochemical variables is an important area for future AI model development, ideally through explicit conditioning on environmental parameters or integration with experimental datasets.

The rapid adoption of AI-predicted models in biomedical research also raises important ethical and practical considerations. Overreliance on low-confidence or unvalidated structural models in drug discovery campaigns, for example, risks misguiding compound design or screening, potentially leading to wasted resources or misleading biological conclusions. It is critical for practitioners to interpret AI-generated confidence metrics cautiously, validate key findings with experimental data where possible, and transparently communicate uncertainty. Community standards and guidelines for responsible AI structure use are needed to ensure scientific integrity and reproducibility (Akdel et al., 2022).

AI techniques have also been applied to predict the conformational ensembles and transient structural propensities of intrinsically disordered proteins (IDPs), rather than fixed, static atomic structures, advanced methods such as NMR spectroscopy and integrative computational modeling are employed. These approaches characterize the range of accessible conformations and dynamic behavior in solution, reflecting the intrinsic heterogeneity that distinguishes IDPs from structured proteins (Jensen et al., 2013; Uversky, 2019). In 2022, RoseTTAFold predicted the structures of neurodegenerative disease-related proteins such as tau and α-synuclein, shedding new light on the molecular basis of protein aggregation and its role in Alzheimer’s and Parkinson’s diseases (Šali and Blundell, 1993). Similarly, in 2023, AlphaFold-Multimer predicted the structure of the SARS-CoV-2 spike protein in complex with human ACE2, revealing key interactions relevant for therapeutic development (Baek et al., 2021).

Deep learning-based approaches such as AlphaFold 2 (AF2) have transformed protein structure prediction by leveraging attention-based neural networks to achieve near-experimental accuracy for globular proteins (Jumper et al., 2021). Its performance in CASP14 established a new benchmark, particularly for monomeric protein folds (Figure 3). However, AlphaFold 3 (AF3) represents a major step forward, capable of predicting not just protein structures but also protein–protein, protein–nucleic acid, and protein–ligand interactions using a unified framework. AF3 integrates structural, chemical, and biophysical constraints, enabling the modeling of functional assemblies and molecular recognition events (Fang et al., 2025). The capabilities of AF3 are summarized in Table 1, distinguishing currently supported applications from prospective claims.

**TABLE 1:** Scope of AlphaFold3 Predictions: Current vs Emerging Capabilities.

Interaction type	Currently supported/ Released	Prospective/ Claimed (emerging)	Example tasks	Expected reliability	Data Prerequisites/ Notes	References
Protein–protein	Yes (AlphaFold-Multimer; extended in AF3)	Improved handling of larger assemblies	Homo-/hetero-dimer modeling; small protein complexes	Benchmarks show ∼60–70% correct interfaces in heterodimers (CASP15, Evans et al. (2022))	Requires deep MSA coverage; higher accuracy with evolutionary coupled pairs	Evans et al. (2022), Kryshtafovych et al. (2023)
Protein–nucleic acid (DNA/RNA)	Limited benchmarks; claimed in AF3 framework	Ongoing validation, not yet robust in CASP	Nucleosome-bound proteins; RNA-binding protein–RNA complexes	Early reports suggest variable accuracy; weaker for flexible RNA	Needs paired MSAs/templates; sparse evolutionary data limits accuracy	Nature Methods commentary, 2024; Kryshtafovych et al. (2023)
Protein–ligand/small molecules	Announced in AF3 white paper; not yet robustly benchmarked	Binding-site modeling and docking	Predict bound cofactors or metabolites	Considered “emerging”; qualitative binding pocket prediction but poor quantitative docking	Requires ligand chemistry input; lacks large curated ligand training datasets	DeepMind AF3 release notes, 2024
Post-translational modifications (PTMs)	Not directly supported	Future claim for handling phosphorylation, glycosylation, etc.	Modeling phosphorylation-dependent conformational changes	Currently unsupported; external tools required	PTM-aware datasets not yet integrated	Jumper et al. (2021), Akdel et al. (2022)

**FIGURE 4:**
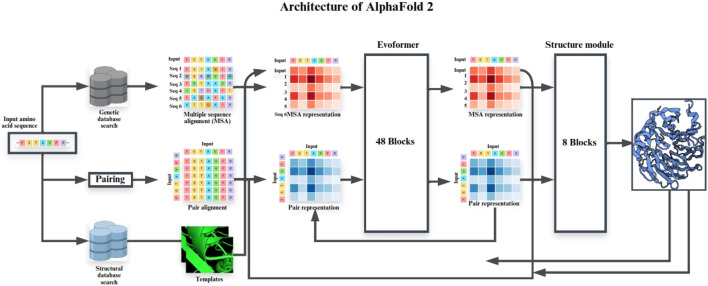
Schematic representation of the AlphaFold 2 architecture for protein structure prediction.

**FIGURE 5:**
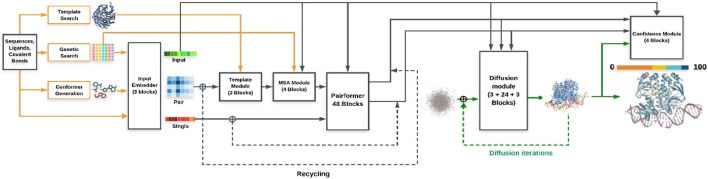
Schematic representation of the AlphaFold 3 architecture for protein structure prediction.

AlphaFold2 (AF2) employs an attention-based architecture with two core components: the MSA track, which captures evolutionary covariation, and the pair representation track, which encodes residue–residue geometry. These interact iteratively through the Evoformer module, and the Structure Module outputs 3D coordinates. This design explains AF2’s breakthrough accuracy in CASP14, achieving near-atomic resolution for most globular proteins. AlphaFold3 (AF3) extends the framework by incorporating multimodal inputs (protein sequences, nucleic acids, ligands) and graph-based chemical embeddings, enabling unified modeling of protein–protein, protein–nucleic acid, and protein–ligand interactions. While still emerging, AF3 benchmarks show improved complex prediction, though ligand-binding remains qualitative. Figures 4, 5 schematically illustrate the AF2 Evoformer–Structure Module pipeline (Figure 4) and AF3’s expanded multimodal framework (Figure 5).

The contribution of AI-driven structure prediction to structural biology has been profound. By providing accurate models for challenging targets such as IDPs and large complexes, AI has complemented experimental approaches and expanded the scope of structural biology. AI has also accelerated drug discovery processes, enabling researchers to identify potential drug targets and design new drugs with greater efficiency. As AI continues to evolve, it will play an increasingly important role in structural biology, opening new directions for future research.

While AI-based predictions have shown transformative accuracy, their responsible use requires careful interpretation of confidence metrics, cross-validation with experimental data, and awareness of pitfalls such as over-interpretation or training leakage. These considerations are summarized in Box 1: Responsible Use of AI-Predicted Structures. Despite their transformative impact, AI-based protein structure prediction tools remain susceptible to reproducibility and generalizability challenges, primarily due to potential biases and limitations in the training datasets. Recent benchmarking efforts have revealed that models like RoseTTAFold and AlphaFold2 may produce less reliable or less reproducible predictions for protein families, domains, or assemblies that are underrepresented in current structural databases. This highlights the ongoing need for rigorous benchmarking, transparency in reporting, and continued expansion and curation of training data sets (Akdel et al., 2022; Kryshtafovych et al., 2023).

### 3.3 Integrative and hybrid methods

Integrative and hybrid methods combine data from multiple techniques, such as cryo-EM, NMR, and small-angle X-ray scattering (SAXS), to build comprehensive models of macromolecular structures. These approaches are particularly useful for studying complex and heterogeneous systems that cannot be fully characterized using a single technique (Maeshima et al., 2014). By integrating data from multiple sources, researchers can generate detailed models that provide a complete picture of macromolecular structures in their biological contexts.

One of the most significant applications of integrative methods has been in the study of the nuclear pore complex (NPC), a massive molecular assembly that regulates the transport of molecules between the nucleus and the cytoplasm. An integrative model of the NPC was reconstructed using cryo-EM, NMR, and biochemical data, providing unprecedented insights into its structure and function (Schotte et al., 2003). This model revealed the molecular mechanisms underlying the NPC’s ability to control transport across the nuclear envelope.

Integrative methods have also been used to study chromatin structure, the complex of DNA and proteins that packages the genome. In 2023, cryo-EM, SAXS, and computational modeling were combined to investigate the arrangement and dynamics of chromatin, shedding light on how chromatin structure regulates gene expression and epigenetic regulation (Mosalaganti et al., 2022).

Mass spectrometry (MS) has emerged as a key structural tool that complements traditional and advanced techniques in structural biology. Approaches such as cross-linking mass spectrometry (XL-MS), native MS, and hydrogen–deuterium exchange MS provide critical information on the architecture, stoichiometry, dynamics, and interaction networks of macromolecular assemblies often in conditions closer to physiological environments (Lenz et al., 2021; Sinz, 2018). When integrated with cryo-EM, NMR, or computational modeling, MS-derived constraints facilitate interpretation of ambiguous regions and help to characterize systems challenging for high-resolution methods alone.

The impact of integrative and hybrid approaches on structural biology has been significant. By enabling the study of complex systems that cannot be fully characterized using a single technique, these methods have expanded the scope of structural biology and provided new tools for addressing challenging biological questions. They have also provided new insights into the dynamic nature of macromolecular biology, revealing the molecular mechanisms underlying complex processes such as gene expression and cellular signaling.

BOX 1 **Responsible use of AI-predicted structures.**

AI-based tools such as AlphaFold2 (AF2) and AlphaFold3 (AF3) have accelerated structural biology, but responsible use requires caution. Below are practical guidelines for researchers and drug discovery practitioners.

**Confidence metrics**

- pLDDT thresholds:
  - >90 → very high confidence (atomic-level reliability).
  - 70–90 → moderate confidence (secondary structure generally correct, side chains less certain).
  - <70 → treat cautiously (often disordered or misfolded).
- PAE (Predicted aligned error):
  - <5 Å → reliable domain/domain positioning.
  - >10 Å → relative orientations uncertain, especially in multi-domain or multimeric complexes.

**Cross-validation with experiments**

- XL-MS: checks residue–residue distances.
- HDX-MS: validates flexible or protected regions.
- SAXS: confirms overall shape/dimensions.
- Mutagenesis & biochemical assays: test pocket or interface predictions.

**IDPs and flexible regions**

- AI models of intrinsically disordered proteins (IDPs) represent hypotheses, not fixed structures.
- Use ensemble-based approaches (e.g., IDPConformerGenerator, ALBATROSS) + NMR/smFRET for realistic modeling.

**Pitfalls to avoid**

- Training leakage: well-studied proteins may appear artificially “predicted.”
- Over-interpretation: confident scores can still misplace domains or interfaces.
- Environmental neglect: AF2/AF3 predictions usually ignore pH, ions, ligands, PTMs.

**✓ Checklist for drug discovery applications**

Before applying AF2/AF3 models in drug discovery, ensure:

- Binding pocket pLDDT >80.
- Cross-validation with mutagenesis, soaking, or NMR CSPs.
- PAE maps confirm correct inter-domain geometry.
- Comparison with orthogonal data (SAXS, cryo-EM, crystallography, homology).
- Flexible loops/IDRs are not solely used for druggability.
- Iterative refinement with MD, docking, MM/GBSA is performed.

### 3.4 Time-resolved structural biology

The term “time-resolved techniques” in structural biology encompasses a diverse set of experimental approaches that operate over distinct temporal scales, each revealing different types of molecular motion or reactivity. For example, time-resolved X-ray crystallography methods including serial femtosecond crystallography at X-ray free-electron lasers (XFELs) capture atomic and electronic changes on the femtosecond to picosecond (10^−15^–10^−12^s) timescale, enabling visualization of ultrafast chemical reactions and bond rearrangements. By contrast, time-resolved cryo-EM is typically limited to millisecond to second (10^−3^–1s) resolution, which is suitable for observing large conformational changes, domain motions, or assembly/disassembly processes in complex macromolecular machines. Techniques such as NMR relaxation and stopped-flow spectroscopies probe dynamics from microseconds to seconds, enabling characterization of backbone motions, folding, or ligand binding events. Because these time scales differ by many orders of magnitude, their applications to biological questions and their technical implementations are fundamentally distinct (Chapman et al., 2011; Fischer et al., 2015; Schanda and Brutscher, 2005).

Time-resolved structural biology aims to capture macromolecular processes in real-time, such as enzyme catalysis, protein folding, and conformational transitions. Techniques such as time-resolved crystallography, time-resolved cryo-EM, and ultrafast spectroscopy allow researchers to visualize macromolecules in motion and understand the molecular mechanisms underlying complex biological events (Aitken et al., 2008). Although time-resolved methods promise to reveal ultrafast dynamics, current implementation in TR-crystallography and TR-cryo-EM remain bounded by mixing, initiation, and data collection constraints. For example, Tenboer et al. (Tenboer et al., 2014) used serial femtosecond crystallography on microcrystals of photoactive yellow protein (PYP), initiating the reaction with light and resolving intermediates to 1.6 Å at the 10 ns to 100 ms timescale using XFEL pulses (Tenboer et al., 2014)this demonstrates what TR crystallography can achieve under ideal conditions. In cryo-EM, recent work using a PDMS-based microfluidic chip achieved tens of milliseconds mixing and freezing, enabling capture of early reaction intermediates though at lower resolution and with stringent demands on sample prep and grid quality (Bhattacharjee et al., 2023). These studies highlight major bottlenecks: reaction initiation speed, reproducible grid manufacture, dose control, and sample thickness.

One of the most significant examples of time-resolved structural biology is the study of the photosynthetic reaction center. In recent years, time-resolved crystallography and serial femtosecond X-ray crystallography (TR-SFX) have been used to map the sequence of electron transfer and water oxidation events in photosystem II. For example, Li et al. (2021) captured the S_1_→S_2_ transition at ambient temperature, revealing water movements and channel rearrangements, while Bhowmick et al. (2023) provided snapshots of the S_3_→[S_4_]→S_0_ transition including ligand rearrangement, the Mn_4_CaO_5_ cluster geometry, and proton/electron release pathways offering critical insight into the final steps of O_2_ formation. Li et al. (2024) revealed structural dynamics of photosystem II during S-state transitions tracking water insertion, proton release, and O-O bond formation that deepen our understanding of photosynthetic energy conversion and inform designs of artificial photosynthetic systems.

Time-resolved cryo-EM has also been used to study GPCR signaling. In 2024, conformational changes in GPCRs during ligand binding and subsequent G protein activation were resolved using time-resolved cryo-EM, providing new insights into the molecular mechanism of signal initiation by GPCRs (Papasergi-Scott et al., 2024). Beyond the application to GPCR conformational dynamics, cryo-EM has enabled visualization of intermediate states in diverse systems. Milestones include sub-second capture of ribosomal translocation steps (Fischer et al., 2015), post-DNA packaging conformational transitions in viral connector/portal complexes (Orlov et al., 2022) and remodeling during capsid maturation (Li et al., 2023). These studies showcase time-resolved cryo-EM’s expanding capacity for mapping transient, functionally relevant conformations in previously inaccessible targets.

The impact of time-resolved structural biology on our understanding of biological processes has been profound. By capturing processes in real-time, these techniques have provided unprecedented insights into the molecular mechanisms underlying complex biological events. They have also informed the development of new drugs and biomaterials, opening new avenues for future research.

### 3.5 Emerging techniques

In addition to the methodologies discussed above, several emerging techniques are expanding the scope of structural biology. These include single-molecule microscopy, mass photometry, and correlative light and electron microscopy (CLEM). These tools provide new ways to study macromolecular structures and dynamics, offering unprecedented insights into biological processes. While single-molecule microscopy and CLEM are now widely adopted in cell biology and have a substantial methodological pedigree, they remain “emerging” within the mainstream of high-resolution, integrative structural biology, where incorporation into multi-modal workflows and large-scale studies is still expanding (Beck and Baumeister, 2016; Hoffman et al., 2020). Recent technical advances continue to broaden their impact and accessibility for complex biological investigations.

Single-molecule microscopy has been used to probe the motions of individual proteins and nucleic acids during translation. For example, in 2023 a smFRET study monitored inter-subunit rotation of ribosomes during translation which contributes to our understanding of how ribosomal dynamics relate to folding and function (Das et al., 2023). This technique is particularly useful for studying intrinsically disordered proteins and their interactions with ligands and binding partners.

Mass photometry is a technique that measures the mass of individual molecules in solution, providing insight into assembly and stoichiometry of macromolecular complexes. For example, in 2021 mass photometry was applied to *SpCas12f1* and *AsCas12f1* CRISPR effectors to resolve their stepwise assembly: first as apo-protein, then forming binary complexes with guide RNA, and then ternary complexes with target DNA (Bigelyte et al., 2021). This technique is powerful for studying protein-nucleic acid interactions and complex assembly (Asor and Kukura, 2022).

Correlative light and electron microscopy (CLEM) combines the spatial resolution of electron microscopy with the specificity of fluorescence microscopy. In recent years, CLEM has been successfully used to study the structure and function of mitochondria in mammalian cells, revealing the dynamic arrangement of cristae and their interactions with other organelles (Jung and Mun, 2019). CLEM is a powerful tool for studying cellular organelles and macromolecular complexes in their native environments. Optical super-resolution microscopy techniques such as STED, PALM, and STORM have enabled fluorescence imaging at nanometer-scale precision. Their integration with electron microscopy through correlative light and electron microscopy (CLEM) workflows known as super-resolution CLEM (SR-CLEM) has become a powerful approach in structural biology. These workflows allow the molecular specificity of fluorescence labeling to be directly correlated with the ultrastructural resolution of EM, enabling the detailed localization of proteins within native cellular environments. Early conceptual development of SR-CLEM highlighted its promise for bridging the resolution gap between light and electron microscopy (de Beer et al., 2023), and later applications, such as the work by Joosten et al. (2018), demonstrated its utility in mapping subcellular features like dendritic cell podosomes. Most recently, Marshall et al. (2023) emphasized how advances in multimodal probes and cryo-fixation technologies have expanded the versatility and resolution of CLEM, making it indispensable for dynamic structural investigations *in situ*. Although super-resolution microscopy can be used independently for structural insights, its primary value in structural biology is as part of multimodal CLEM studies that bridge the resolution gap between molecular imaging and EM ultrastructure.

The impact of these emerging techniques on structural biology has been profound. By providing new ways to study complex biological structures, these tools have expanded the scope of structural biology and opened exciting new avenues for future research. The strengths and limitations of various structural characterization techniques are summarized in Table 2.

**TABLE 2:** Overview of major structural biology techniques.

Technique	Description	Strengths	Limitations	References
X-ray Crystallography	Determines atomic structures by analyzing diffraction of X-rays from crystallized macromolecules	Highest resolution (≤1.2 Å possible); mature and widely available; critical for drug discovery (e.g., SARS-CoV-2 Mpro, GPCRs)	Requires high-quality crystals; unsuitable for many flexible/membrane proteins; suffers from the “phase problem.”	Kendrew et al. (1958), Rasmussen et al. (2011), Zhang et al. (2020), Smith et al. (2023)
NMR Spectroscopy	Uses magnetic resonance of nuclei to study macromolecules in solution, revealing structure and dynamics	Captures proteins in near-native environments; excellent for conformational dynamics, ligand binding, and IDPs	Size limitations (<40–50 kDa typically); requires isotope labeling and high-field instruments; complex analysis	Wüthrich (2001), Ostrem et al. (2013), Burré et al. (2014), Tycko (2011)
Cryo-Electron Microscopy (Cryo-EM)	Visualizes vitrified biomolecules using direct electron detectors and advanced image processing	No crystallization required; ideal for large complexes, membrane proteins, and flexible systems; resolutions ∼3 Å common, best near 1.2 Å	High cost; demanding sample prep and computational infrastructure; requires CTF correction	Kühlbrandt (2014), Liao et al. (2013), Fitzpatrick et al. (2017), Iudin et al. (2023), Singh et al. (2024)
AI-Driven Prediction	Uses deep learning (e.g., AlphaFold2/3, RoseTTAFold) to predict structures from sequence, often at atomic accuracy	Rapid, global accessibility; high accuracy for globular proteins; supports large-scale proteome modeling	Limited for IDPs, multi-protein complexes, and novel folds; dependent on evolutionary data; ignores environment (pH, ligands, PTMs)	Jumper et al. (2021), Varadi et al. (2022), Kryshtafovych et al. (2023), Lotthammer et al. (2024)
Mass Spectrometry (MS)	Measures mass/charge of biomolecules (native MS, XL-MS, HDX-MS) for structural/topological analysis	Provides stoichiometry, topology, and dynamics; integrates well with EM/NMR/SAXS; works near-physiological conditions	Lower resolution; specialized reagents and analysis required	Sinz (2018), Lenz et al. (2021)
Integrative Methods	Combines multiple data sources (e.g., cryo-EM, NMR, SAXS, MS, AI) to build hybrid models	Captures large, flexible, heterogeneous complexes; leverages complementary data	Complex data integration; variable/local resolution	Kim et al. (2018), Mosalaganti et al. (2022)
Time-Resolved Techniques	Captures molecular dynamics with femtosecond crystallography, time-resolved cryo-EM, ultrafast spectroscopy, or NMR.	Visualizes transient states and conformational transitions in real time; functional insight	Limited by equipment (XFELs, fast freezing); technique-specific temporal resolution	Tenboer et al. (2014), Bhattacharjee et al. (2023), H. Li et al. (2021), Bhowmick et al. (2023)
Emerging Techniques	Includes single-molecule fluorescence, mass photometry, correlative light and EM (CLEM), cryo-ET.	Enables *in situ* visualization and dynamics at cellular scale; powerful for native context studies	Lower resolution; technically demanding; still expanding in structural biology	Beck and Baumeister (2016), Das et al. (2023), Bigelyte et al. (2021), Marshall et al. (2023)

## 4 Applications of high-resolution structural characterization

The development of advanced techniques for characterizing macromolecular structures has transformed our ability to study biological processes at the molecular level. These advancements have facilitated breakthroughs in drug discovery, enzymology, nanotechnology, and the study of disease mechanisms. By providing atomistic insights into the structure and function of proteins, nucleic acids, and other macromolecules, these techniques have enabled the development of new therapeutics, engineered enzymes, biomaterials, and a deeper understanding of disease at the molecular level.

### 4.1 Drug discovery

In drug discovery, structure-based drug design (SBDD) is a cornerstone of modern pharmacology. By leveraging high-resolution structures of macromolecules, researchers can identify binding pockets and design high-affinity, high-efficacy inhibitors. For example, the X-ray crystallographic determination of the BRAF kinase structure revealed the molecular mechanism of its activation in melanoma, leading to the development of vemurafenib, a targeted therapy for BRAF-mutant melanoma (Long et al., 2014). More recently, cryo-EM was used to resolve the structure of HER2 kinase in complex with a novel inhibitor, paving the way for next-generation therapies for HER2-positive breast cancer (Bornscheuer et al., 2012).

While structure-based drug design (SBDD) and AI-driven platforms have accelerated the identification of therapeutic candidates, several real-world limitations persist. For example, although SBDD enabled the development of vemurafenib for BRAF V600E-mutant melanoma, rapid emergence of resistance driven by MAPK pathway reactivation or alternative mutations highlights the ongoing gap between structural prediction and durable clinical efficacy (Long et al., 2014). Similarly, commercial AI drug design efforts, such as Isomorphic Labs’ reported pipelines, face challenges including dependency on the depth and diversity of training datasets, potential model bias, and limited interpretability of predictions, with few compounds yet reaching advanced clinical stages (Walters and Murcko, 2020). Finally, although cryo-EM has expanded opportunities for structure-guided development of biologics like antibodies, the field faces scalability constraints due to high equipment costs, specialized labor, and throughput bottlenecks in sample preparation and data processing, which currently limit high-volume industrial application (Mao, 2025; Punjani et al., 2017; Renaud et al., 2018).

Structural biology has also played a critical role in antiviral drug development. The structure of the SARS-CoV-2 main protease (Mpro), determined using cryo-EM and X-ray crystallography, enabled the design of nirmatrelvir, an antiviral therapeutic that inhibits viral replication (Owen et al., 2021; Zhang et al., 2020). Similarly, NMR spectroscopy has been used to characterize the dynamics of HIV-1 protease, leading to the development of a new class of drugs with reduced drug resistance (Zeltins, 2013). These advancements have accelerated the development of targeted therapies, reducing the cost and time required for drug discovery while improving efficacy and reducing side effects. The integration of AI-driven structure prediction tools, such as AlphaFold, has further enhanced these efforts by providing high-fidelity models of drug targets and their interactions with small molecules (Jumper et al., 2021).

### 4.2 Enzymology

In enzymology, structural characterization has enabled the engineering of enzymes for industrial applications, including biofuel production, waste management, and pharmaceutical manufacturing. For example, high-resolution X-ray structures of cellulases have directly enabled enzyme engineering for biofuels: structure-guided recombination and crystallography of GH5 endoglucanases produced chimeras with higher thermostability and activity (Chang et al., 2016), and comparative/structure-based redesign of GH7 cellobiohydrolases improved processive activity on cellulose (Taylor et al., 2018). Although cryo-EM is increasingly applied to thermostable enzymes, definitive cryo-EM studies linking loop stabilization to simultaneous gains in cellulase stability and catalysis are not yet established; instead, cryo-EM has provided high-resolution models for other thermostable enzymes, underscoring feasibility for future cellulase work (Sobhy et al., 2022).

Lipases, industrially important enzymes used in detergents and pharmaceuticals, have been engineered using structural and dynamics insights. Rather than a single NMR-only mapping study, recent work combines structural analysis with computation to target flexible/functional regions (e.g., lids/tunnels) and improve specificity or stability: a 2023 review documents successful computer-aided lipase engineering strategies spanning structure prediction, docking, and MD-guided mutagenesis (Cheng and Nian, 2023). For operation in organic media, CALB variants were evaluated in organic solvent with combined experiments and computation; mutations near the active site reduced sensitivity to water activity and preserved activity in non-aqueous conditions (Tjørnelund et al., 2023). Mechanistic QM/MM MD on CALB further clarified how environment (pH) and catalytic geometry govern reactivity and regioselectivity principles that guide rational variant design (Świderek et al., 2023). Together, these studies show that integrative, computation-informed engineering (rather than AI alone) is currently the most evidenced path to lipases with broadened specificity and improved performance in challenging solvent systems.

Hemoglobin has been well resolved by cryo-EM alone, but no peer-reviewed studies have yet demonstrated a direct AI–cryo-EM synergy in its structural analysis. In contrast, cytochrome P450 has been examined by combining AlphaFold2 predictions with cryo-EM density maps to explore alternative conformational states, underscoring how AI can complement experimental data (Urban and Pompon, 2022). To better represent the scope of AI in cryo-EM, more robust examples include the Fanconi anemia core complex, where deep learning predictions enabled *de novo* model building into a 4.6 Å cryo-EM map that was otherwise uninterpretable (Farrell et al., 2020), CryoDRGN, which applied generative neural networks to uncover continuous conformational heterogeneity in ribosomes and spliceosomes (Zhong et al., 2021), and the nuclear pore complex, where AlphaFold2-derived models filled unresolved regions in cryo-EM maps to achieve near-complete reconstruction of this ∼120 MDa. Together, these cases provide clearer evidence of how AI directly enhances cryo-EM, from improving model building to revealing hidden structural states.

### 4.3 Nanotechnology

The design of nanodevices and biomaterials derived from macromolecular structures has opened new frontiers in nanotechnology. For example, cryo-EM and crystallographic studies of ferritin nanocages have enabled the development of ferritin-based nanoparticles for targeted drug delivery and imaging, exploiting their highly stable cage architecture and ability to encapsulate diverse cargos (Lee et al., 2022; Mohanty et al., 2022). Viral-like particles (VLPs) are another emerging class of nanomaterials: structural studies of bacteriophage Qβ VLPs have clarified their assembly pathways and stability (Shaw et al., 2022),and such scaffolds are now being investigated as potential platforms for mRNA vaccine delivery (Lin et al., 2024). Structural insights have also accelerated progress in biosensing. The crystallographic determination of glucose oxidase provided the basis for the first highly sensitive blood glucose biosensors in diabetes care, while more recent NMR analyses of glucose oxidase variants revealed conformational dynamics that guided the design of improved, durable biosensors (Alatzoglou et al., 2023; Mahamid et al., 2016).

In tissue engineering, structural studies have informed the design of biomaterials for regenerative medicine. The cryo-EM determination of the structure of collagen has enabled the development of collagen scaffolds for tissue regeneration (Neutze et al., 2000). In 2021, an integrative model of the interaction between collagen and fibronectin led to the development of a biomaterial for wound healing (Frantz et al., 2010). These advancements have transformed medicine and materials engineering, enabling the development of new drugs, diagnostics, and regenerative therapies.

### 4.4 Understanding disease mechanisms

Structural characterization has provided critical insights into the molecular basis of disease, facilitating the development of targeted therapies and diagnostics. For example, NMR determination of the structure of the prion protein revealed the molecular basis of its misfolding in prion diseases such as Creutzfeldt-Jakob disease (Prusiner, 1998). Cryo-EM resolution of misfolded tau protein structures has shed light on their role in neurodegenerative diseases (Fitzpatrick et al., 2017), while cryo-EM determination of amyloid-β fibrils has guided therapeutic strategies targeting aggregation in Alzheimer’s disease (Gremer et al., 2017). Recently, solid-state NMR revealed the structure of α-synuclein fibrils, offering insights into their toxicity in Parkinson’s disease (Tuttle et al., 2016). Beyond elucidating fibril morphologies, high-resolution cryo-EM structures of tau aggregates have directly facilitated rational PET tracer design with improved selectivity for disease-associated folds. For example, the conformation-specific features of AD tau fibrils guided the development and optimization of PET ligands such as [18F] PI-2620, enhancing early diagnosis and monitoring of tauopathies (Fitzpatrick et al., 2017; Kroth et al., 2019).

Structural studies have also played a critical role in combating viral infections. The cryo-EM determination of the SARS-CoV-2 spike protein structure revealed the molecular basis of viral entry into host cells, guiding the development of vaccines and antiviral therapies (Wrapp et al., 2020). In 2021, cryo-EM resolved the structure of the Ebola virus glycoprotein, informing the development of a novel antiviral drug (Lee et al., 2008). These advancements have revolutionized our understanding of complex diseases, enabling the development of targeted therapies and diagnostics. The integration of AI-driven structure prediction and integrative modeling has further enhanced our ability to study disease mechanisms and develop effective treatments (Jumper et al., 2021).

Structural biology has revolutionized drug discovery, enabling the development of targeted therapies such as vemurafenib for BRAF-mutant melanoma and nirmatrelvir for COVID-19 (Table 3). Recent research has demonstrated the power of structural biology in addressing global health challenges. For example, the structure of the SARS-CoV-2 main protease, determined using Cryo-EM and X-ray crystallography, enabled the rapid development of nirmatrelvir, an antiviral drug for COVID-19 (Table 4).

**TABLE 3:** Applications of structural characterization.

Application	Techniques used	Examples	Impact	References
Drug Discovery	X-ray crystallography, Cryo-EM, NMR, AI tools	Structure of SARS-CoV-2 Mpro → design of nirmatrelvir	Accelerated rational drug design; targeted therapies	Zhang et al. (2020), Owen et al. (2021), Renaud et al. (2018)
Enzymology	X-ray crystallography, Cryo-EM, NMR, computation/AI	Structure-guided cellulase engineering (GH5/GH7); lipase redesign with computation/QM/MM	Improved industrial enzymes; mechanism insight	Chang et al. (2016); (GH7 redesign, Taylor et al., 2018, as discussed); Cheng and Nian (2023), Świderek et al. (2023)
Nanotechnology	Cryo-EM, X-ray/NMR, computational modeling	Ferritin nanocages for delivery; mRNA-vaccine VLP/nano carriers overview	Novel drug carriers & biosensing platforms	Lee et al. (2022), Lin et al. (2024)
Disease Mechanisms	Cryo-EM, solid-state NMR, NMR, integrative modeling	Tau filaments (AD); Aβ(1–42) fibrils; SARS-CoV-2 spike	Structural basis for pathology; PET tracers; vaccines/antivirals	Fitzpatrick et al. (2017), Gremer et al. (2017), Wrapp et al. (2020), Prusiner (1998)

**TABLE 4:** Seminal contributions and recent advances in structural biology.

Technique	Study	Findings/Breakthrough	Impact/Application	References
Cryo-EM	Tau filaments from Alzheimer’s brain (Fitzpatrick et al., 2017)	First patient-derived atomic structure of tau filaments	Revealed disease-specific folds; guided tau PET tracer development	Fitzpatrick et al. (2017), Kroth et al. (2019)
	SARS-CoV-2 spike protein (Wrapp et al., 2020)	First full-length 3D structure of coronavirus spike	Enabled rational vaccine and therapeutic design during COVID-19	Wrapp et al. (2020)
Cryo-EM + Integrative Modeling	Nuclear pore complex (Kim et al., 2018; Mosalaganti et al., 2022)	Full architecture of NPC	Unified multimethod approach; megadalton assemblies	Kim et al. (2018), Mosalaganti et al. (2022)
X-ray Crystallography	β2-Adrenergic receptor (Rasmussen et al., 2011)	First high-resolution GPCR structure	Revolutionized GPCR drug discovery; revealed activation mechanism	Rasmussen et al. (2011)
	KcsA potassium channel (Doyle et al., 1998)	First ion channel atomic structure	Explained ion selectivity; launched ion channel biophysics	Doyle et al. (1998)
Time-Resolved Crystallography	Cytochrome c oxidase (Tenboer et al., 2014; H. Li et al., 2021; Bhowmick et al., 2023)	Captured femtosecond–millisecond electron/proton transfer	Validated ultrafast SFX; illuminated respiratory catalysis	Tenboer et al. (2014), H. Li et al. (2021), Bhowmick et al. (2023)
AI Prediction	AlphaFold Protein Structure Database (Jumper et al., 2021; Varadi et al., 2022)	Predicted nearly entire human proteome	Democratized structural biology; accelerated functional annotation	Jumper et al. (2021), Varadi et al. (2022)
NMR Spectroscopy	Prion protein (Riek et al., 1996)	First atomic structure of a prion protein	Provided structural basis of prion diseases	Riek et al. (1996)
Solid-state NMR	α-synuclein fibrils (Tuttle et al., 2016)	High-resolution fibril structures	Advanced understanding of Parkinson’s pathology	Tuttle et al. (2016)
Hybrid/AI-assisted Cryo-EM	Fanconi anemia core complex; CryoDRGN (Farrell et al., 2020; Zhong et al., 2021)	AI-aided model building and conformational heterogeneity	Expanded cryo-EM interpretability and heterogeneity analysis	Farrell et al. (2020), Zhong et al. (2021)

## 5 Challenges and future directions

Notwithstanding significant advances in structural characterization techniques, a range of challenges still impedes our complete understanding of macromolecular structure and function in living matter. Overcoming them will require new thinking and new technology creation. In parallel, integration of new tools, such as artificial intelligence (AI) and machine learning, holds out hope for widening horizons in structural biology. In the following, we review significant obstacles and future trends in the field.

### 5.1 Overcoming challenges in membrane proteins and intrinsically disordered proteins

Membrane proteins and intrinsically disordered proteins (IDPs) remain among the most difficult targets in structural biology due to their inherent dynamics, instability, and, for membrane proteins, pronounced obstacles in crystallization and sample preparation. Recent years have witnessed notable advances that address these barriers. For membrane proteins, innovations in lipidic cubic phase (LCP) crystallization, including the introduction of novel lipids such as 7.10 MAG, have significantly expanded the range of targets amenable to in meso crystallization and improved success rates in obtaining high-resolution structures (Krawinski et al., 2024). In parallel, methods such as the VIALS approach now enable the high-throughput generation of dense microcrystals for serial crystallography, streamlining the structural analysis of challenging membrane proteins (Birch et al., 2023). The rapid adoption of single-particle cryo-EM with improved detectors and computational tools has also led to a dramatic increase in the number and resolution of membrane protein structures, now routinely achieving resolutions better than 3 Å for complex assemblies (Thangaratnarajah et al., 2022).

The field of IDP research has similarly advanced through new experimental and computational methodologies. High-throughput platforms based on cell-free protein crystallization (CFPC) are enabling rapid structure determination and systematic assessment of factors that stabilize or modulate IDP conformational ensembles (Bastida et al., 2023; Kojima et al., 2024). Analytical methods that combine molecular dynamics, single-molecule FRET, and advanced NMR spectroscopy now routinely characterize the residue-level dynamics and binding-competent conformations of disordered regions, illuminating their mechanisms in signal transduction and disease (Bastida et al., 2023; Orand and Jensen, 2025). In addition, “proteomimetic” scaffolds and targeted biophysical screens are being developed to selectively trap or modulate functional IDP conformations, opening new avenues for therapeutic targeting of these previously intractable proteins (Nguyen et al., 2025). Collectively, these innovations continue to break down longstanding technical barriers, deepening our understanding of these essential biomolecules in health and disease.

### 5.2 Integrating structural information with functional and dynamical analysis

Whereas macromolecular structure reveals significant information regarding macromolecular topology, a complete understanding of function requires integration of information regarding structure, function, and dynamics. Most processes in living cells, such as enzyme catalysis, transmembrane signal transduction, and protein folding, entail complex conformational processes, whose complete description cannot be achieved with static structures alone. Time-resolved techniques, such as time-resolved crystallography and cryo-EM, have begun to circumvent such difficulty by capturing snapshots of processes in motion at an atomic level. In 2010, time-resolved crystallography was used to study the catalytic cycle of cytochrome c oxidase, mapping the sequence of electron and proton transfers during oxygen reduction (Kaila et al., 2010). Recent advances in single-molecule FRET have also illuminated protein folding dynamics, yielding new insights into macromolecular interactions (Hartmann et al., 2023; Schuler et al., 2016).

### 5.3 Expanding the role of AI and machine learning in structure prediction and refinement

The use of AI and machine learning in structural biology has already seen a transformational impact, with examples including the success of AlphaFold and RoseTTAFold in predicting structures with high accuracy (Jumper et al., 2021). Not only have these tools accelerated the pace of structure discovery, but access to structures has democratized, and researchers worldwide can explore the molecular basis of life. In 2021, AlphaFold achieved high accuracy in predicting the structures of folded proteins, but its performance on IDPs and macromolecular complexes remained limited (Ruff and Pappu, 2021). In 2023, machine learning methods such as EMReady were applied to cryo-EM density maps to refine local and non-local features and significantly improve map-model correlation and interpretability, especially for maps in the 3–6 Å resolution range (He et al., 2023).

### 5.4 Developing new technologies for in situ structural characterization

One of the most challenging and exciting frontiers in structural biology is developing tools for studying macromolecules in their native environment within living cells. Most conventional methodologies in structural biology require isolating and purifying macromolecules, a manipulation that can distort function and structure. *In situ* characterization of structure, in which macromolecules are analyzed in whole cells, holds out hope for overcoming such restrictions and providing a truer view of the environment in life. Recent advances in cryo-electron tomography (cryo-ET) have made it possible to visualize macromolecular machines directly in cells, providing critical insights into their native organization. Although cryo-ET delivers molecular or mesoscale detail rather than near-atomic resolution, most cellular tomograms are reconstructed at 3–8 nm, limited by lamella thickness, signal-to-noise ratios, and radiation damage. Sub-nanometer resolution in intact cells is not yet realistic, although subtomogram averaging can improve detail for abundant complexes. Recent advances such as focused ion beam (FIB) milling for lamella thinning and Volta phase plates for contrast enhancement have further improved data quality. For example, Balyschew et al. (Balyschew et al., 2023) demonstrated ∼3 Å resolution for favorable targets using FIB-milled lamellae and subtomogram averaging, while Berger et al. (2023) showed how automated FIB workflows facilitate higher-resolution tomography in crowded environments. Fung et al. (2023) applied genetically encoded tags with AI-based detection to improve protein localization, and Zhou and Lok (2024) highlighted both the promise and limits of visualizing viral assembly *in situ*. Together, these studies underscore that atomic detail is largely restricted to isolated complexes or averaged subtomograms, while whole-cell reconstructions provide mesoscale structural insights.

Recent advances in correlative light and electron microscopy (CLEM) have enabled detailed visualization of mitochondrial ultrastructure and dynamic interactions with other organelles in mammalian cells, linking molecular specificity to the *in situ* ultrastructural context (Jiang et al., 2022; Jung and Mun, 2024). Progress moving forward will depend on developing new modalities such as super-resolution CLEM workflows and improved embedding/fiducial labeling protocols and enhancing algorithms for registration, segmentation, and interpretation of complex *in situ* data (Franek et al., 2024; Jung and Mun, 2024).

## 6 Standards and reproducibility

Reproducibility is the cornerstone of structural biology, ensuring that discoveries made through experimental cryo-electron microscopy (cryo-EM) and computational AI-based predictions can be independently verified, critically assessed, and reused for future research. Both domains have developed community-driven standards that focus on mandatory data deposition, rigorous validation, and transparent reporting.

### 6.1 Cryo-EM standards

Over the past decade, cryo-EM has matured into a method with strong archiving policies and widely adopted validation practices. To promote transparency, it is now mandatory that cryo-EM reconstructions be deposited in community repositories: density maps in the Electron Microscopy Data Bank (EMDB), atomic coordinate models in the Protein Data Bank (PDB), and, increasingly, raw micrographs and particle stacks in the Electron Microscopy Public Image Archive (EMPIAR) (Iudin et al., 2023; Lawson et al., 2024). Together, these archives provide the full spectrum of data from raw experimental images to refined atomic models enabling reproducibility and method development. The growth of EMPIAR, with over 1,000 datasets deposited by 2023, has been particularly important for benchmarking particle picking, developing new algorithms, and validating reconstructions in areas such as membrane protein biology (Kleywegt et al., 2024).

Validation remains a central issue. The Fourier Shell Correlation (FSC), particularly the gold-standard half-map FSC using the 0.143 criterion, is the most widely used metric for estimating resolution and identifying overfitting (Rosenthal and Henderson, 2003). However, FSC only provides a global average, so local resolution assessment is now standard practice, using tools such as ResMap and Blocres to capture variations across flexible or heterogeneous regions (Ku. Model–map fit is evaluated with global correlation coefficients, per-atom inclusion scores, and more targeted tools such as EMRinger, which assesses the agreement of side-chain rotamers with density (Barad et al., 2015). FSC-Q, a local correlation-based metric, has further refined map–model validation by addressing the limitations of global FSC in anisotropic or unevenly resolved maps (Afonine et al., 2018; Pintilie and Chiu, 2021).

Community initiatives have formalized these practices. The 2019 EMDataResource Model Challenge highlighted that no single validation metric is sufficient; instead, reproducibility requires a combination of complementary assessments across map quality, model geometry, and map–model agreement (Lawson et al., 2021). Building on this, the 2024 EM Ligand Modeling Challenge extended reproducibility standards to ligand-bound complexes, recommending not only the evaluation of ligand density fit but also systematic validation of geometry, stereochemistry, and binding-pocket environment (Lawson et al., 2024). These efforts illustrate how validation has moved beyond reporting resolution alone to providing a multi-dimensional quality assessment.

Finally, reporting checklists have been adopted to standardize practices across laboratories. The EM Validation Task Force (VTF) (Henderson et al., 2012) called for mandatory reporting of half-map FSC curves, map sharpening strategies, and overfitting tests. Later wwPDB/EMDB workshops codified these into structured checklists, including microscope parameters, data-processing pipelines, validation reports, and deposition metadata (Lawson et al., 2021). Such measures align with broader reproducibility frameworks in biomedical research, such as the EQUATOR Network, adapted for structural biology to ensure completeness and reduce bias.

### 6.2 AI-based model standards

AI-based predictions, particularly from AlphaFold2 (AF2), AlphaFold-Multimer, and more recently AlphaFold3, have expanded structural biology beyond the experimental Frontier. However, their reproducibility depends on input transparency. To replicate a model, researchers must release the exact multiple sequence alignments (MSAs), template sets, and random seeds used during inference (Jumper et al., 2021; Mirdita et al., 2022). Even small differences in alignment depth or stochastic seeds can alter the outcome, especially for disordered or multi-domain proteins. Without sharing these inputs, predictions become non-reproducible “black boxes.”

Confidence metrics are integral to reproducibility. pLDDT (predicted Local Distance Difference Test) scores provide per-residue confidence estimates, while Predicted Aligned Error (PAE) matrices quantify uncertainty in relative domain or chain positioning (Jumper et al., 2021). Publishing these metrics alongside structural coordinates allows other researchers to interpret which regions are reliable and which require caution. Empirical studies show that high pLDDT values (>90) generally correlate with accurate folds, but low-scoring regions often correspond to intrinsic disorder, requiring ensemble modeling or experimental validation (Ruff and Pappu, 2021).

Cross-validation with experimental methods is also essential. Techniques such as small-angle X-ray scattering (SAXS), hydrogen–deuterium exchange mass spectrometry (HDX-MS), and cross-linking MS (XL-MS) can confirm or refute AI-predicted folds and interactions. Benchmarking exercises like CASP15 demonstrated that while AlphaFold performs exceptionally for stable, globular proteins, reproducibility declines for multi-protein complexes, novel folds, and environments not represented in training data, underscoring the importance of complementary experimental validation (Kryshtafovych et al., 2023).

As with cryo-EM, community repositories are beginning to support AI models. Large-scale predictions are already available through the AlphaFold Protein Structure Database and the ESM Atlas, while individual researchers are encouraged to deposit AI-generated structures in ModelArchive or PDB-Dev with accompanying metadata. This ensures transparency, supports benchmarking, and prevents selective reporting of only “successful” predictions.

### 6.3 Integrative perspective

Standards in cryo-EM and AI are converging toward a shared framework: mandatory deposition of primary data, use of multiple complementary validation metrics, and transparent reporting of inputs and methods. While cryo-EM emphasizes archival and validation pipelines, AI-based predictions stress input transparency and confidence scoring. Together, these practices ensure that both experimental and computational structures can be trusted, compared, and built upon by the community, ultimately fostering more robust and integrative approaches to protein modeling.

## 7 Conclusion

The field of structural biology has undergone a revolution over the past several decades, driven by advances in imaging, computational modeling, and the integration of experimental and computational approaches. From the early days of X-ray crystallography to the modern era of cryo-electron microscopy (cryo-EM) and AI-driven structure prediction, these techniques have provided unprecedented insights into the structure and function of biological macromolecules. By revealing the atomic details of proteins, nucleic acids, and other macromolecular assemblies, structural biology has not only deepened our understanding of fundamental biological processes but has also facilitated breakthroughs in drug discovery, enzymology, nanotechnology, and the study of disease mechanisms.

The advent of cryo-EM, with its ability to visualize large macromolecular complexes and membrane proteins at near-atomic resolution, has democratized structural biology, making it accessible to researchers worldwide. Similarly, AI tools such as AlphaFold and RoseTTAFold have revolutionized the field by enabling the prediction of protein structures with remarkable accuracy and speed. Integrative and hybrid approaches, which combine data from multiple techniques, have expanded the scope of structural biology, providing new tools for studying complex biological systems and revealing the dynamic nature of macromolecular interactions.

These advancements have had a profound impact on drug discovery, enabling the development of targeted therapies with high efficacy and specificity. From anticancer drugs to antiviral therapies for COVID-19, structural biology has guided the development of life-saving treatments. In enzymology, structural insights have enabled the engineering of enzymes with desired properties, transforming industrial processes and driving innovation in biotechnology. In nanotechnology, structural studies have informed the design of nanodevices and biomaterials, opening new avenues for drug delivery, biosensing, and tissue engineering. Structural characterization has also provided critical insights into the molecular basis of disease, enabling the development of targeted therapies and diagnostics for conditions such as Alzheimer’s disease, Parkinson’s disease, and viral infections.

Despite these remarkable achievements, significant challenges remain. Membrane proteins and intrinsically disordered proteins (IDPs) continue to pose technical challenges, and new methodologies will be needed to stabilize and characterize these complex systems. Integrating structural, functional, and dynamical data will be essential for achieving a complete understanding of biological processes, and continued refinement of time-resolved and integrative techniques will be critical. The expansion of AI and machine learning tools holds great promise for improving the accuracy and efficiency of structure prediction and refinement, but further work is needed to extend these tools to more complex systems.

Looking to the future, the development of new technologies for *in situ* structural characterization offers exciting opportunities to study macromolecules in their native cellular environments. These advancements have the potential to provide a more accurate and comprehensive view of biological processes, driving further innovation in structural biology and its applications.

In conclusion, structural biology will continue to play a central role in advancing our understanding of life at the molecular level. By integrating new technologies, computational approaches, and multidisciplinary methodologies, researchers will be able to tackle increasingly complex biological questions, opening new frontiers in science and technology. From dissecting disease mechanisms to developing new drugs and bio-inspired materials, structural biology will remain at the heart of scientific progress, shaping the future of biology and medicine.
